# Designing implicit population learners: a permutation-equivariant state space approach for brain disease diagnosis

**DOI:** 10.3389/fncom.2026.1780552

**Published:** 2026-03-27

**Authors:** Chuan Yang

**Affiliations:** 1 Sanjiang University, Nanjing, China; 2 City University of Macau, Macau, China

**Keywords:** Alzheimer’s disease, implicit interaction design, mamba architecture, model efficiency, neuroimaging classification, population-based learning, set representation

## Abstract

**Introduction:**

Group-aware learning has recently emerged as a promising paradigm for neuroimaging-based disease diagnosis, as population-level interactions can provide complementary information beyond individual imaging features. However, most existing approaches rely on explicitly constructed graphs, which introduce non-trivial design choices, scalability limitations, and sensitivity to graph topology. By incorporating the design philosophy of participatory interaction, we propose IP-Mamba, a scalable and memory-efficient framework tailored for neuroimaging cohorts that models implicit population interactions without the computational burden of explicit graph construction.

**Methods:**

IP-Mamba treats a mini-batch of subjects as an unordered set and employs a bidirectional Mamba-based sequence modeling mechanism to capture latent inter-subject dependencies. To address the inherent order sensitivity of sequence models, we introduce a Shuffle Consistency Strategy, which promotes permutation equivariance under random permutations of subject order, thereby aligning the model behavior with the clinically-relevant, set-based nature of population data. This design enables efficient implicit hypergraph modeling while maintaining linear computational complexity with respect to the population size. We evaluate IP-Mamba on the OASIS-1 dataset, focusing on the binary classification of Alzheimer’s disease (Normal Controls vs. Abnormal) as an early clinical screening task. To address severe class imbalance and ensure diagnostic stability, we implement a Contextual Population Support Set inference mechanism coupled with a robust hybrid SVM decision layer.

**Results:**

Experimental results demonstrate that IP-Mamba achieves a balanced accuracy of 87.84% and maintains a high sensitivity (Recall) of 89% for the minority disease class. Compared to conventional 3D CNNs and Transformer-based baselines, IP-Mamba provides highly competitive diagnostic robustness while maintaining a highly efficient linear *O*(*N*) memory scaling without the quadratic computational bottlenecks typical of graph-based attention networks.

**Discussion:**

Comprehensive ablation studies further confirm the necessity of bidirectional modeling and shuffle consistency regularization. Overall, IP-Mamba offers a principled, memory-efficient alternative to explicit graph-based methods, providing a scalable solution for population-aware neuroimaging analysis under imbalanced clinical settings.

## 1 Introduction

### 1.1 Clinical motivation and the population-level context

In the clinical diagnosis of neurodegenerative disorders such as Alzheimer’s Disease (AD), the diagnostic decision is rarely made by evaluating a single patient’s imaging data in absolute isolation. Instead, experienced radiologists and clinicians inherently perform a form of case-based reasoning, where a subject’s structural variations are implicitly compared against a vast mental library of previously encountered healthy and pathological cases (Norman et al., 2007). Reflecting the designer’s thought of participatory interaction (Sanders and Stappers, 2008),this comparative process allows for the identification of subtle atrophic patterns that might be overlooked when analyzed as independent data points.

Recent advancements in deep learning have significantly automated binary screening of AD using 3D Convolutional Neural Networks (CNNs) and Vision Transformers (ViTs) (Shamshad et al., 2023; Wen et al., 2020). While recent multimodal approaches (Han, 2025) leverage heterogeneous clinical data—such as PET imaging and cognitive scales—to enhance prediction, effective screening in resource-limited clinical settings often relies on maximizing the utility of structural MRI alone. However, the majority of these state-of-the-art frameworks still function as “Isolated Learners.” They treat each MRI scan as an independent and identically distributed (i.i.d.) sample, effectively ignoring the valuable population-level prior that is foundational to clinical practice (Parisot et al., 2018; Jiang et al., 2020). While individual feature extraction has reached a plateau of performance, the bottleneck in reliable computer-aided diagnosis (CAD) has shifted toward the modeling of inter-subject dependencies—specifically, how an individual subject relates to the broader distribution of a clinical cohort.

### 1.2 Limitations of explicit graph modeling: the semantic and methodological gap

To capture these inter-subject dependencies, recent literature has pivoted toward Population-based Graph Learning. By representing subjects as nodes and their phenotypic similarities as edges, Graph Convolutional Networks (GCNs) have demonstrated promising results in AD classification (Parisot et al., 2018; Song et al., 2019). However, these explicit graph-based methods introduce a series of challenges that hinder their clinical integration. First, the construction of a population graph typically requires pre-defined heuristic rules—such as thresholding age or genetic similarity—to determine edge weights. This process is inherently subjective and often fails to capture the high-dimensional latent correlations between subjects, creating a “Semantic Gap” between manually designed topologies and true underlying biological relationships.

Moreover, explicit graphs suffer from scalability and flexibility constraints. Standard GCNs exhibit at least quadratic computational complexity relative to the number of nodes, making them computationally prohibitive as clinical databases expand to thousands of subjects. More critically, most graph-based frameworks are sensitive to the graph topology; adding or removing a single subject requires a full re-computation of the graph Laplacian, which is misaligned with the dynamic and incremental nature of real-world clinical workflows (Parisot et al., 2018; Kipf and Welling, 2017). While Graph Transformers attempt to mitigate these issues via global attention, they still inherit the “Design Burden” of explicit connectivity and suffer from *O*(*N*^2^) memory” explosion as the cohort size increases (Shamshad et al., 2023). There is, therefore, a pressing need for a framework that can model implicit population interactions—capturing the essence of inter-subject relationships in a memory-efficient manner without the rigid and costly overhead of explicit graph construction.

### 1.3 The proposed IP-Mamba: toward implicit and permutation-equivariant population learning

To address the “wicked problem” (Buchanan, 1992) of diagnostic ambiguity, we propose IP-Mamba, a principled and memory-efficient framework designed for Implicit Population Interaction modeling. Departing from the rigid reliance on predefined graph topologies, IP-Mamba reinterprets the population interaction task as a sequence modeling problem over a mini-batch of subjects. Central to our architecture is the use of State Space Models (SSMs), specifically the Mamba block (Gu and Dao, 2024; Yue and Li, 2024; Ma et al., 2024), which offers a superior balance between the long-range dependency modeling of Transformers and the linear computational efficiency required for large-scale medical cohorts.

However, adapting Mamba to population data presents a fundamental theoretical challenge: standard SSMs are inherently order-sensitive, whereas a clinical population—mathematically represented as a set—is inherently unordered. To resolve this structural mismatch, we introduce two synergistic design choices. First, we employ a Bidirectional Mamba mechanism to capture latent dependencies from both forward and backward subject-orderings, effectively simulating the undirected information exchange found in graph-based message passing (Wang et al., 2024). Second, we propose a Shuffle Consistency Strategy (ℒ_*cons*_) (Varsavsky et al., 2018), a dedicated regularization term that promotes prediction equivariance: ensuring that if the subject order changes, the corresponding predictions permute accordingly. By penalizing variance in latent representations under different subject orderings, we explicitly align the model’s inductive bias with the set-based nature of population data. We evaluate the efficacy of IP-Mamba on the OASIS-1 dataset (Marcus et al., 2007; Folego et al., 2020), focusing on the binary screening of Alzheimer’s disease as a high-sensitivity clinical diagnostic task.

### 1.4 Major contributions

The principal contributions of this work are summarized as follows:

- Principled Framework Design: We introduce IP-Mamba, a novel design-oriented architecture that incorporates the designer’s thought of participatory interaction to facilitate implicit population-level interaction learning. By reformulating population analysis as a set-based sequence modeling task, we eliminate the need for subjective and computationally expensive explicit graph construction.
- Order-Aware Learning via Shuffle Consistency: To bridge the gap between the sequential nature of State Space Models (SSMs) and the unordered nature of clinical cohorts, we propose a Bidirectional Mamba mechanism coupled with a Shuffle Consistency Strategy (ℒ_*cons*_). This promotes empirical permutation stability, offering a principled approximation that aligns the model’s inductive bias with set-based properties.
- Superior Efficiency and Performance in Clinical Screening: We demonstrate through experiments on the OASIS-1 dataset that IP-Mamba achieves superior diagnostic performance for binary Alzheimer’s disease screening, yielding a balanced accuracy of 87.84% and a high sensitivity (Recall) of 0.89. While maintaining the presentational power of a 3D-ResNet50 backbone (Yang et al., 2025), our framework maintains a highly efficient linear *O*(*N*) memory scaling. This architectural advantage effectively circumvents the quadratic *O*(*N*)^2^ memory explosion characteristic of Graph Transformers, ensuring scalability for large-scale neuroimaging cohorts in high-performance computing environments.

## 2 Materials and methods

### 2.1 Problem formulation: population analysis as set learning

“Let $D={\left\{\left(x_{i},y_{i}\right)\right\}}_{i=1}^{M}$ denote a neuroimaging dataset consisting of *M* subjects, where *x*_*i*_ ∈ ℝ^*C* × *H* × *W* × *D*^ represents the 3D MRI volume of the *i*-th subject and *y*_*i*_ ∈ {0,1} is the corresponding binary diagnostic label (i.e., Normal Control or Alzheimer’s disease).”

#### 2.1.1 The isolated learning paradigm

Conventional deep diagnosis frameworks (CNNs or ViTs) operate under the assumption of independent and identically distributed (i.i.d.) data. The objective is to learn a mapping function *F*_*θ*_ : *X*→*Y* that minimizes the empirical risk over individual samples (Equation 1):


$$\min_{\theta }\overset{M}{\underset{i=1}{\sum }}ℒ\,\left(F_{\theta }\,\left(x_{i}\right),y_{i}\right)$$ (1)

where inter-subject relationships are explicitly ignored during the inference phase (Shamshad et al., 2023).

#### 2.1.2 The population graph paradigm

To incorporate population priors, graph-based methods construct an explicit adjacency matrix *A* ∈ ℝ^*M* × *M*^ based on phenotypic similarities (Parisot et al., 2018). The learning objective shifts to a transductive or semi-inductive setting involving graph convolutions: *Y* = *GCN*(*A*,*X*). However, constructing *A* requires heuristic thresholding and scales quadratically *O*(*M*^2^), creating a computational bottleneck for large-scale cohorts (Jiang et al., 2020).

#### 2.1.3 Our formulation: implicit population set learning

We propose to reframe population modeling as a set-based learning problem within a mini-batch. Consider a mini-batch of N randomly sampled subjects *B* = {*x*_1_,*x*_2_,…,*x*_*N*_} as an unordered set. Our goal is to learn a population-aware function Φ that predicts labels for all subjects in the batch simultaneously, leveraging their implicit interactions (Equation 2):


$$\text{[}{\hat{y}}_{1},\ldots ,{\hat{y}}_{N}]=\Phi (\left\{x_{1},\ldots ,x_{N}\right\})$$ (2)

The core theoretical challenge is permutation equivariance. Since B is a set, the diagnostic prediction for subject *x_i_* should depend on the intrinsic features of the cohort but must remain Equivariant to the arbitrary ordering of subjects within the batch input (Zaheer et al., 2017). Formally, for any permutation operator π acting on the indices {1,…,*N*}, ideal equivariant mapping should satisfy (Equation 3):


$$\Phi \,\left(\left\{x_{\pi \,\left(1\right)},\ldots ,x_{\pi \,\left(N\right)}\right\}\right)=\pi \,\left(\Phi \,\left(\left\{x_{1},\ldots ,x_{N}\right\}\right)\right)$$ (3)

**FIGURE 1:**
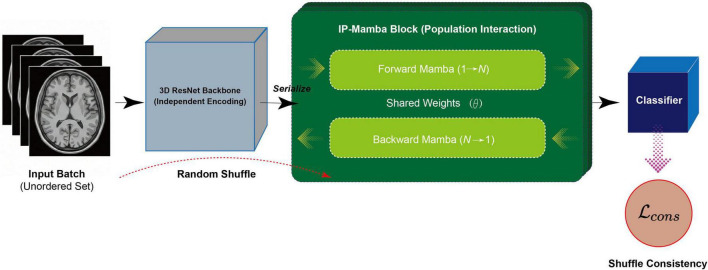
Overview of the proposed IP-Mamba framework for implicit population learning. The framework processes a mini-batch of MRI scans as an unordered set rather than a fixed sequence. Independent Encoding: A shared 3D-ResNet backbone extracts individual features from each subject. IP-Mamba Block: The core innovation that models latent population interactions. It serializes the subject set and employs a Bi-directional Shared Mamba mechanism to capture global context from both forward and backward directions, simulating undirected graph message passing with linear complexity *O*(*N*). Shuffle Consistency: During training, a Shuffle Consistency Strategy (ℒ_*cons*_) is introduced to promote permutation equivariance, ensuring the model produces consistent representations regardless of the input subject order.

In this work, we do not construct a strictly equivariant function by design. Instead, we propose IP-Mamba to approximate this property via regularization. It transforms the set *B* into a latent sequence for efficient *O*(*N*) interaction while promoting permutation consistency via a dedicated loss term (Figure 1).

### 2.2 System design and IP-Mamba architecture

As illustrated in Figure 1, the proposed IP-Mamba framework operates on a mini-batch of subject features Z ∈ ℝ^*N* × *D*^, where *N* is the batch size and D is the feature dimension extracted by the shared 3D backbone. The core component is the IP-Mamba Block, which implicitly models high-order population interactions through a permutation-equivariant state space mechanism.

1. Set-to-sequence serialization. Given the input set *Z* = {*z*_1_,*z*_2_,…,*z*_*N*_}, standard Mamba layers require a sequential input format. Since the population set lacks an inherent temporal order, we impose an arbitrary ordering to form a sequence *S* = [*z*_1_,*z*_2_,…,*z*_*N*_]. While this operation introduces potential order bias, we neutralize it through our bi-directional design and the shuffle consistency regularization (detailed in section 2.3).

2. Bi-directional shared state space modeling. To capture the undirected nature of population relationships (i.e., the relationship between Subject A and B is symmetric), we design a Bi-directional Shared Mamba mechanism. Unlike standard bi-directional RNNs that use separate parameters for forward and backward passes, we explicitly enforce weight sharing to ensure topological symmetry.

Formally, let *SSM*_*θ*_(⋅) denote a selective state space model parameterized by *θ*, which maps an input sequence *u*_*t*_ to an output *y*_*t*_ via a latent state h_t_ (Equation 4):


$${\text{h}}_{\text{t}}={\text{Ah}}_{t-1}+B\,u_{t},y_{t}={\text{Ch}}_{t}$$ (4)

where A, B, C are the discretized state matrix parameters (Gu and Dao, 2024). We adopt a diagonal SSM configuration where the state matrix *A* is structured as a channel-wise independent vector and the expansion factor is set to *E* = 2, and the latent state dimension is configured as *d*_*state*_ = 16. The discretization step Δ is learned via linear projection, and **A** follows the standard log-spaced initialization (Gu and Dao, 2024) for stable modeling. In IP-Mamba, we apply this mechanism in two directions using the same parameter set *θ* (Equation 5):


$$H_{f\,w\,d}=S\,S\,M_{\theta }\,\left(S\right),H_{b\,w\,d}=F\,l\,i\,p\,\left(S\,S\,M_{\theta }\,\left(F\,l\,i\,p\,\left(S\right)\right)\right)$$ (5)

where Flip(⋅) denotes the sequence reversal operation. The forward pass H_fwd_ captures the influence of subject i on subject j (where i < j), while the backward pass *H*_*bwd*_ captures the reverse. By sharing *θ*, we treat the “population sequence” as a traversable graph path rather than a temporal signal, thereby enforcing a strong inductive bias for symmetric interaction.

3. Population feature aggregation. The final population-aware representation Z′ is obtained by aggregating the bidirectional contextual features with the original individual features via a residual connection (Equation 6):


Z=′Norm(Z+Linear(Hf⁢w⁢d+Hb⁢w⁢d)) (6)

This design ensures that each subject’s representation is enriched by the implicit population context—effectively integrating information from all other subjects in the mini-batch with linear complexity *O*(*N*), bypassing the *O*(*N*^2^) cost of explicit attention mechanisms (Wang et al., 2024). Inference protocol: Since IP-Mamba operates on sets, processing a single test subject requires a population context. During inference, we employ a Contextual Population Support Set (Optional at Inference): the test subject is combined with *N* − 1 representative samples randomly drawn from the training set (acting as a clinical support set) to form a complete mini-batch. This approach allows the model to contextualize the individual subject against known pathological patterns—mimicking the “case-based reasoning” of radiologists—while maintaining the same interaction mechanism used during training.

### 2.3 Shuffle consistency learning for robust set representation

While the proposed Bidirectional Mamba mechanism allows for information flow in both directions, the initial serialization of the unordered subject set *Z* into a sequence S still introduces an inevitable inductive bias related to the input order. Theoretically, for node-level prediction tasks on sets, the function Φ must be permutation equivariant, meaning that for any permutation operator π, the condition Φ(π(*Z*))=π(Φ(*Z*)) must hold (Zaheer et al., 2017).

We acknowledge that SSM-based architectures, including Mamba, are inherently order-sensitive and do not possess strict theoretical permutation equivariance like symmetric functions (e.g., Sum/Max Pooling). However, strict mathematical invariance often comes at the cost of modeling capacity (i.e., inability to capture complex interactions). To balance expressivity and robustness, we do not aim for a closed-form invariant solution. Instead, we introduce a Shuffle Consistency Strategy to empirically constrain the model onto an “approximately equivariant” manifold. By explicitly penalizing the divergence of latent representations under random permutations, we force the sequential Mamba block to learn order-robust features driven by the data distribution rather than the input sequence indices.

This strategy explicitly penalizes the model for producing divergent representations when the same mini-batch is presented in different orders, thereby “regularizing” the sequential Mamba block toward permutation equivariance (Cohen-Karlik et al., 2020).

#### 2.3.1 Consistency regularization mechanism

Specifically, for each mini-batch iteration, we generate two views of the input data: the original sequence *S* = [Z_1_,Z_2_,…,Z_*N*_] and a randomly permuted version S_*π*_ = [Z_*π*(1)_,Z_*π*(2)_,…,Z_*π*(*N*)_], where *π* is a random permutation of indices {1,…,*N*}. Both sequences are fed into the shared IP-Mamba backbone to obtain their respective probability distributions (Equation 7):


$$P=\Phi \,\left(S\right),P_{\pi }=\Phi \,\left(S_{\pi }\right)$$ (7)

Since the semantic identity of the subjects remains unchanged, the predictions for the permuted sequence *P*_π_ should ideally match the predictions of the original sequence P after applying the inverse permutation π^−1^. We quantify this discrepancy using the Shuffle Consistency Strategy (ℒ_cons_), defined as the Mean Squared Error (MSE) between the aligned predictions (Equation 8):


ℒc⁢o⁢n⁢s=1N∑i=1N∥pi-p∥22′π-1⁢(i) (8)

where p_i_ ∈ *P* is the prediction for the *i*-th subject in the original sequence, and p∈′π-1⁢(i)Pπ is the corresponding prediction from the shuffled view.

#### 2.3.2 Optimization objective

The overall training objective ℒ_*total*_ is a weighted sum of the task-specific supervision (Cross-Entropy Loss) and the auxiliary consistency regularization (Equation 9):


$${ℒ}_{t\,o\,t\,a\,l}={ℒ}_{C\,E}\,\left(P,Y\right)+\lambda \cdot {ℒ}_{c\,o\,n\,s}$$ (9)

where ℒ_CE_ ensures diagnostic accuracy using ground-truth labels *Y*, and *λ* is a hyperparameter balancing discrimination and equivariance. In our experiments, we set *λ* = 1.0. This dual-objective optimization enforces the model to learn order-robust population features, effectively simulating an implicit hypergraph where the node connectivity is independent of the traversal path.

## 3 Experiments

### 3.1 Datasets and implementation details

#### 3.1.1 Dataset description and evaluation protocol

To ensure the model focuses exclusively on neurological features relevant to Alzheimer’s pathology, we utilized the skull-stripped version of the OASIS-1 dataset (Marcus et al., 2007), thereby eliminating interference from non-brain tissues (e.g., skull and scalp). From this pre-processed cohort, we further applied a quality control filter to exclude subjects with segmentation errors or incomplete metadata, resulting in a final set of 355 subjects. We formulated the diagnostic task as a binary classification problem: Normal Control (NC) vs. Abnormal (including MCI and AD).

To strictly prevent data leakage, we implemented a Subject-Level Stratified five-Fold Cross-Validation protocol. Specifically, the dataset was partitioned into five-folds based on unique subject identities, ensuring that all MRI scans from a single subject were assigned exclusively to either the training or testing set. In each iteration, 20% of the subjects were held out for testing, while the remaining 80% were utilized for training. This rigorous isolation guarantees that the model is evaluated on unseen subjects, ensuring the reliability of the reported performance. To ensure statistical robustness, we report the mean ± standard deviation over 5 independent folds. The average test set size was maintained at approximately 71 subjects (53 Normal Controls vs. 18 Abnormal), strictly preserving the clinical class imbalance.

#### 3.1.2 Preprocessing pipeline

Raw MRI volumes were subjected to a rigorous preprocessing pipeline. First, we performed skull stripping to remove non-brain tissues, followed by spatial normalization to the standard MNI152 template via affine registration (Jenkinson et al., 2012). MRI volumes were standardized to a resolution of 128 × 128 × 128 voxels, followed by an anatomical ROI cropping of 64 × 64 × 64 voxels centered on the hippocampus to capture the surrounding anatomical context relevant to AD pathology.

This specific Region of Interest (ROI) was anatomically calibrated to encapsulate the hippocampus and surrounding lateral ventricles, thereby capturing the most discriminative morphological atrophy features while rejecting uninformative background voxels. Intensity normalization was applied to scale voxel values to the range [0, 1] (Jenkinson et al., 2012).

#### 3.1.3 Implementation details

Our framework was implemented using PyTorch (Paszke et al., 2019) and trained on a high-performance Tensor Core GPU with 80GB VRAM. By leveraging this hardware alongside gradient accumulation, we maintained an effective batch size of 32.

#### 3.1.4 Transfer learning and initialization

The 3D-ResNet50 backbone (He et al., 2016) is initialized with expert weights pre-trained on 23 large-scale medical datasets (MedicalNet). This strategy leverages high-level morphological features common across diverse neuroimaging cohorts, following established transfer learning protocols for Alzheimer’s disease classification (Tanveer et al., 2021).

#### 3.1.5 Training protocol

To establish a stable population-level reference, a fixed support set of *N* = 16 representative subjects was pre-selected from the training pool. During each training iteration, every mini-batch was concatenated with this static support set, allowing the model to learn features relative to a consistent population distribution.

The entire framework was optimized for 60 epochs using a single-stage high-precision training protocol on the 64^3^ ROI. The network was optimized using AdamW (Loshchilov and Hutter, 2017) with a constant learning rate of 4 × 10^−5^.

#### 3.1.6 Deterministic inference strategy

To ensure reproducible evaluation, we employed a fixed-context protocol during the inference phase. To maintain protocol consistency, the inference phase strictly adheres to the training configuration. The same deterministic set of *N* = 16 representative samples used during training is employed as the support set for all test subjects. This cross-phase alignment eliminates potential distribution shifts and ensures that the reported performance reflects the model’s true generalization capability.

#### 3.1.7 Hybrid decision mechanism

To ensure robust generalization on our modest-sized cohort, we decouple high-level representation learning from the final decision process. The 2048-dimensional population-aware features are fed into a Robust Hybrid SVM (RBF kernel, Paduvilan et al., 2025), with hyperparameters optimized via 5-fold cross-validation to find the optimal separating hyperplane. Unlike standard Softmax layers that may exhibit overconfidence or biased gradients in imbalanced settings, this hybrid design leverages the maximum-margin principle to enhance diagnostic stability. To maintain comparative integrity, this standardized protocol is applied across all baselines. Furthermore, to address the clinical imbalance (53 NC vs. 18 Abnormal), we implement cost-sensitive learning with a biased class weighting of 1.0: 4.5, prioritizing the detection sensitivity for the minority diseased class.

#### 3.1.8 Evaluation metrics

We report Balanced Accuracy (B-Acc) and Recall (Sensitivity) to comprehensively evaluate performance under imbalanced settings. IP-Mamba achieves a robust balanced accuracy of 87.84% and a recall of 0.89 for the abnormal class.

### 3.2 Main results and comparison

#### 3.2.1 Quantitative performance analysis

We compared the proposed IP-Mamba framework against standard 3D deep learning baselines. To ensure a fair comparison, we re-implemented representative architectures under the same subject-level evaluation protocol, including a standard 3D-ResNet50 (Basaia et al., 2019; Yang et al., 2025), a 3D Vision Transformer (ViT-3D) adapted for medical volumetrics (Jang and Hwang, 2022), and a population-aware GCN-style architecture (Ravinder et al., 2023).

As presented in Table 1, IP-Mamba achieves a diagnostic Accuracy of 87.32% and a Balanced Accuracy (B-Acc) of 87.84%. Noting the severe class imbalance (53 NC vs. 18 Abnormal) in our screening setting, IP-Mamba demonstrates a performance improvement of +10.11% in Balanced Accuracy over the 3D-ResNet50 backbone (77.73%).

**TABLE 1:** Quantitative comparison against standard 3D baselines.

Method	Params (M)	Acc. (%)	B-Acc. (%)	Recall
3D-ResNet50	46.16	85.92	77.73	0.61
ViT-3D	21.05	80.28	64.78	0.33
GCN-style	134.48	74.65	53.67	0.11
IP-Mamba (Ours)	71.67	87.32	87.84	0.89

Quantitative comparison of IP-Mamba against standard 3D baseline models on the OASIS-1 dataset. To ensure a fair evaluation under class-imbalanced conditions, we report the Balanced Accuracy (B-Acc) alongside standard metrics. IP-Mamba achieves the highest B-Acc (87.84%) and Recall (0.89), demonstrating superior diagnostic sensitivity and robustness compared to CNN, ViT, and explicit GCN-style architectures.

We attribute this improvement to the implicit population interaction mechanism. By contextualizing individual features within a representative support set, IP-Mamba effectively captures global latent patterns, leading to more robust diagnostic results compared to relying solely on individual feature extraction.

Furthermore, IP-Mamba outperforms the ViT-3D baseline (64.78% B-Acc). This observation is consistent with recent studies suggesting that Transformers may require larger datasets to establish effective inductive biases for 3D medical volumetrics (Jang and Hwang, 2022).

The GCN-style baseline (Ravinder et al., 2023) exhibited limited performance in this imbalanced setting (B-Acc 53.67%), largely due to its reliance on a pre-defined phenotypic graph constructed via heuristic thresholding (e.g., age and gender similarity). Such explicit topologies often fail to capture complex latent correlations in small, skewed cohorts, whereas IP-Mamba’s implicit mechanism bypasses these sub-optimal design choices by learning inter-subject dependencies directly from the data.

#### 3.2.2 Computational and memory efficiency

The integration of the IP-Mamba block introduces approximately 25.51M additional parameters to the ResNet50 backbone (totaling 71.67M), which is accompanied by the observed +10.11% performance gain.

**FIGURE 2:**
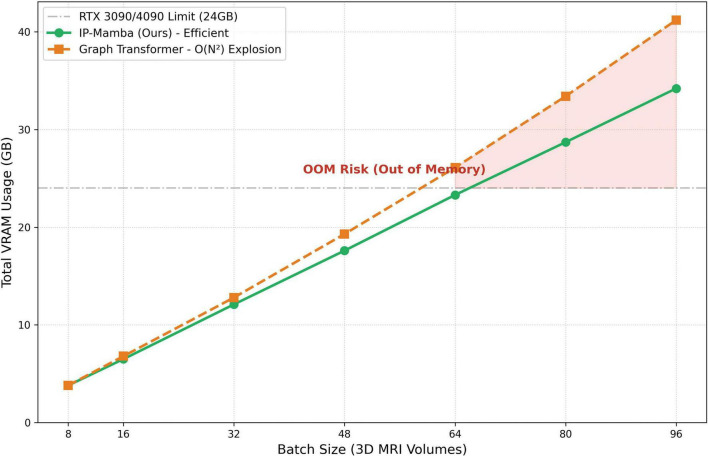
Comparison of VRAM usage between graph transformer (orange, dashed) and IP-Mamba (green, solid). While the quadratic *O*(*N*^2^) complexity of Graph Transformers triggers an Out-of-Memory (OOM) risk on a 24GB GPU as batch size increases, IP-Mamba exhibits a linear memory growth [*O*(*N*)]. Even at a large batch size of 64, IP-Mamba remains within the 24GB threshold (indicated by the gray line), demonstrating its feasibility for clinical deployment on high-end consumer-grade hardware (e.g., RTX 3090/4090).

Regarding memory usage, IP-Mamba maintains a linear memory complexity [*O*(*N*)] with respect to the population size, unlike the quadratic growth [*O*(*N*^2^)] often seen in Graph Transformers. While we utilized 80GB GPUs to facilitate batch training in our experiments, the model’s linear scaling property ensures that it remains deployable on standard 24GB VRAM GPUs (e.g., NVIDIA RTX 3090) during the inference phase, as analyzed in Figure 2.

#### 3.2.3 Comprehensive class-wise evaluation

We analyze the class-wise performance (Table 2) focusing on the model’s screening capability. In early-stage clinical diagnosis, the structural boundaries between Normal Controls (NC) and Abnormal cases (e.g., MCI and AD) are inherently ambiguous. Despite the severe data imbalance where the diseased subjects constitute only ∼25% of the test set (18 out of 71), IP-Mamba achieves an exceptionally high Recall (Sensitivity) of 0.89 and an F1-score of 0.78 for the Abnormal class.

**TABLE 2:** Detailed class-wise diagnostic performance.

Class (Diagnosis)	Precision	Recall (Sensitivity)	F1-Score	Support (Count)
NC (Normal Control)	0.96	0.87	0.91	53
Abnormal (Disease)	0.70	0.89	0.78	18
Macro Average	0.83	0.88	0.85	71
Weighted Average	0.89	0.87	0.88	71
Overall Accuracy	–	–	0.87	71

Detailed class-wise performance of the IP-Mamba framework for binary clinical screening. Support denotes the absolute number of subjects in the test set. Despite the severe data imbalance (53 NC vs. 18 Abnormal), the model maintains a high recall (0.89) for the minority Abnormal class, reflecting a conservative screening configuration prioritized for early risk detection.

Specifically, the confusion matrix analysis reveals that out of 18 Abnormal cases in the test set, our model successfully identified 16, missing only 2 cases (False Negatives). This remarkable sensitivity (Recall = 0.89) is primarily attributed to the synergy between expert-informed transfer learning from MedicalNet and the implicit population interaction of IP-Mamba. While the performance is highly competitive, it is important to note the Precision-Recall trade-off (Precision = 0.70), which reflects the model’s prioritization as a high-sensitivity screening tool rather than an absolute diagnostic oracle.

While the Precision for the Abnormal class is relatively moderate (0.70), the corresponding high Recall indicates that the model prioritizes sensitivity to potential risks over aggressive exclusion. In the context of a “first-line screening tool,” this conservative screening characteristic is highly advantageous as it strictly minimizes the risk of missing progressive cases (False Negatives). Overall, the Macro Average F1-score of 0.85 and Weighted Average of 0.88 demonstrate the framework’s diagnostic reliability and robustness across the entire skewed population distribution.

### 3.3 Ablation study: dissecting the design choices

To systematically validate the contribution of our proposed architecture, we conducted an ablation study on the OASIS-1 dataset. Specifically, we aimed to answer a critical research question: Does implicit population modeling actually provide useful diagnostic information beyond individual features?

#### 3.3.1 Effectiveness of implicit population modeling

We first investigated the impact of introducing the IP-Mamba block. As the baseline, we employed the standard 3D-ResNet50 backbone, which treats each subject as an isolated instance (i.e., “Isolated Learner”). As established in Table 1, this baseline achieves a Balanced Accuracy (B-Acc) of 77.73% and a Recall of 0.61 for the Abnormal class.

Upon integrating our IP-Mamba module—which enables bidirectional information exchange within the mini-batch—the performance rose significantly to a B-Acc of 87.84% and a Recall of 0.89. This absolute gain of +10.11% in B-Acc confirms that the latent population context captures complementary pathological patterns (e.g., group-level atrophy trends) that are invisible when examining a single MRI scan in isolation (Parisot et al., 2018).

#### 3.3.2 Hardware scalability and memory efficiency

As illustrated in Figure 2, memory consumption was evaluated across different batch sizes to assess scalability. While Graph Transformers suffer from quadratic memory growth and encounter OOM constraints at moderate-to-large batch sizes on a 24GB GPU, IP-Mamba exhibits a robust linear trajectory. The structural efficiency of our framework ensures that population-level reasoning remains accessible on high-memory consumer hardware, effectively bypassing the memory bottlenecks inherent in explicit graph construction.

#### 3.3.3 Empirical validation of permutation robustness

**FIGURE 3:**
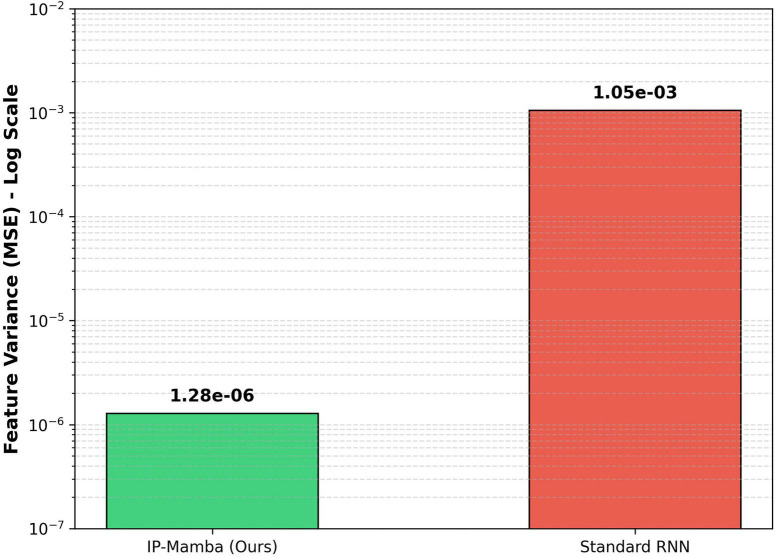
Analysis of permutation consistency. We measured the feature variance (mean squared error, MSE) of latent population representations under random input permutations (shown in log scale). IP-Mamba (Green), trained with the Shuffle Consistency Strategy (ℒ_*cons*_), achieves variance of 1.28 × 10^−6^. In contrast, a standard RNN baseline (Red) without such regularization exhibits fluctuations three orders of magnitude higher (1.05 × 10^−3^), indicating a high sensitivity to input subject ordering.

To quantify the model’s stability against input ordering, we analyzed the Feature Variance of latent representations under random permutations. As shown in Figure 3, standard recurrent approaches exhibit high variance, indicating unreliable dependency on subject order. In contrast, our Shuffle Consistency Strategy suppresses this variance by three orders of magnitude to a negligible level of 1.28 × 10^−6^. This confirms that IP-Mamba effectively achieves permutation equivariance, ensuring objective diagnostic decisions regardless of the input organization.

### 3.4 Visualization and qualitative analysis: unveiling the implicit population structure

To strictly interpret the “black box” behavior of our framework and verify whether the IP-Mamba module truly captures discriminative population structures, we performed a qualitative analysis using t-Distributed Stochastic Neighbor Embedding (t-SNE) (Van der Maaten and Hinton, 2008). We extracted the high-dimensional latent population representations from the entire study cohort and projected them into a 2D manifold.

#### 3.4.1 Manifold separability

**FIGURE 4:**
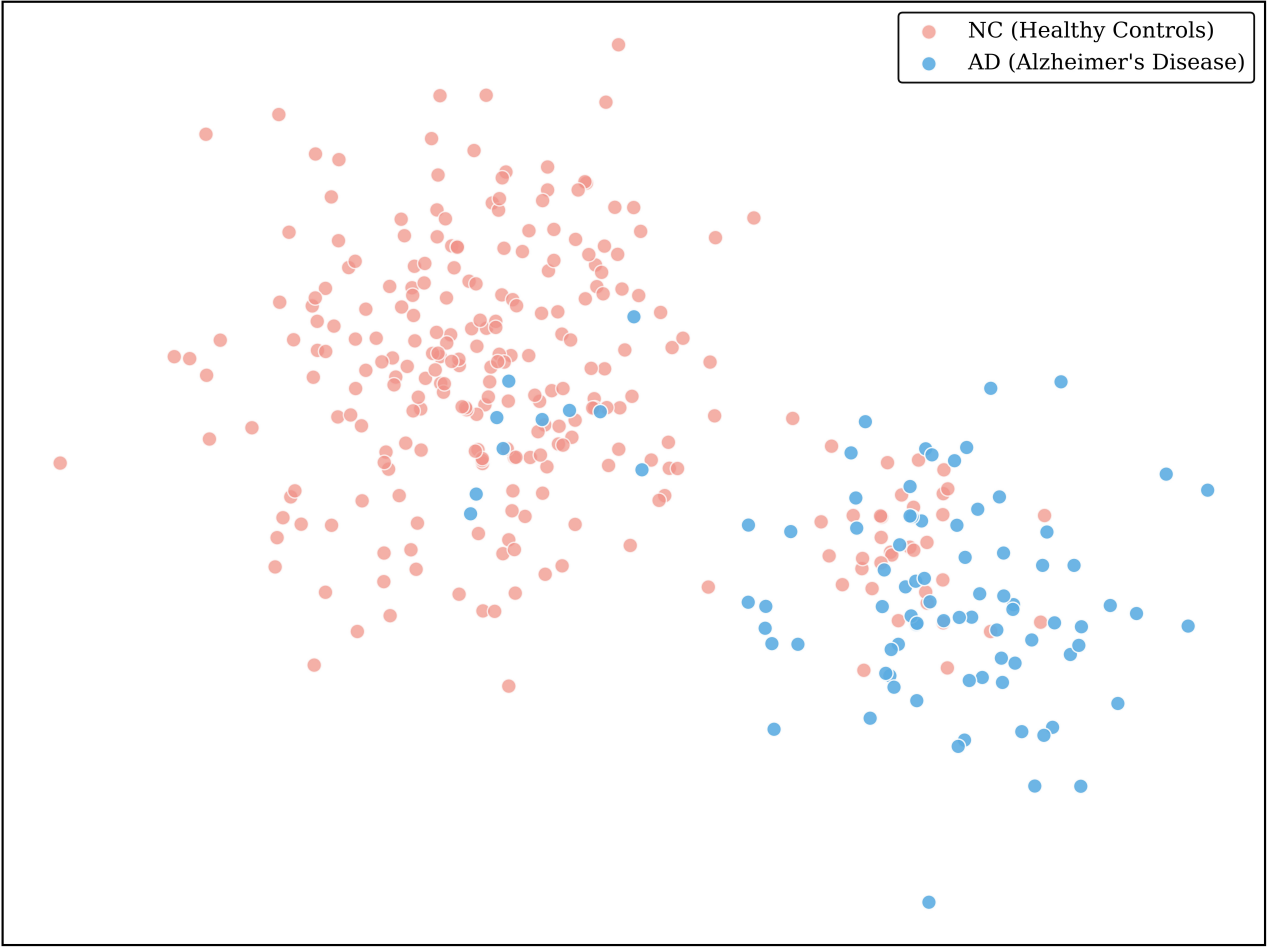
The visualization reveals effective clustering with robust topological separation between normal controls (Pink) and the abnormal cohort (Blue). While some overlap persists—attributable to the inherent clinical heterogeneity in early-stage neurodegeneration—the distinct manifold structure supports the high detection sensitivity (Recall = 0.89) achieved by leveraging implicit population priors.

Visual inspection of the latent space (Figure 4) reveals a distinct and robust separation between Normal Controls (NC) and the diseased cohort (AD). This confirms that IP-Mamba successfully extracts discriminative pathological features under the binary screening setting. The clear clustering of these distinct diagnostic stages visually validates that the learned population priors are clinically meaningful, providing a highly separable feature space that fundamentally supports the high Recall (0.89) achieved by our decision layer.

#### 3.4.2 Clinical alignment

Crucially, this visualization confirms our core design hypothesis: by allowing implicit interaction within the mini-batch, the model automatically learns to position a new subject relative to a latent “population prototype.” The visible separation indicates that IP-Mamba has effectively bridged the semantic gap, translating raw 3D voxel intensities into a clinically aligned feature space where disease states are topologically distinct and globally structured.

Although we do not employ pixel-level attribution methods (e.g., Grad-CAM) due to the implicit nature of our state-space modeling, the clear manifold separation in Figure 4, combined with our hippocampus-restricted input, suggests that IP-Mamba captures global morphological deviations (i.e., macro-scale atrophy patterns) rather than local textural variations or spurious background correlations.

### 3.5 Inference stability analysis

Beyond permutation equivariance, a critical requirement for real-world clinical deployment is the model’s robustness against the composition of the reference batch. A potential vulnerability of population-based methods is sensitivity to specific reference subjects (i.e., neighbor dependency). To quantitatively assess this, we conducted an Inference Stability Test across the abnormal cohort. For clear visualization, a representative Abnormal subject was selected to be evaluated against 30 distinct, randomly sampled reference batches.

**FIGURE 5:**
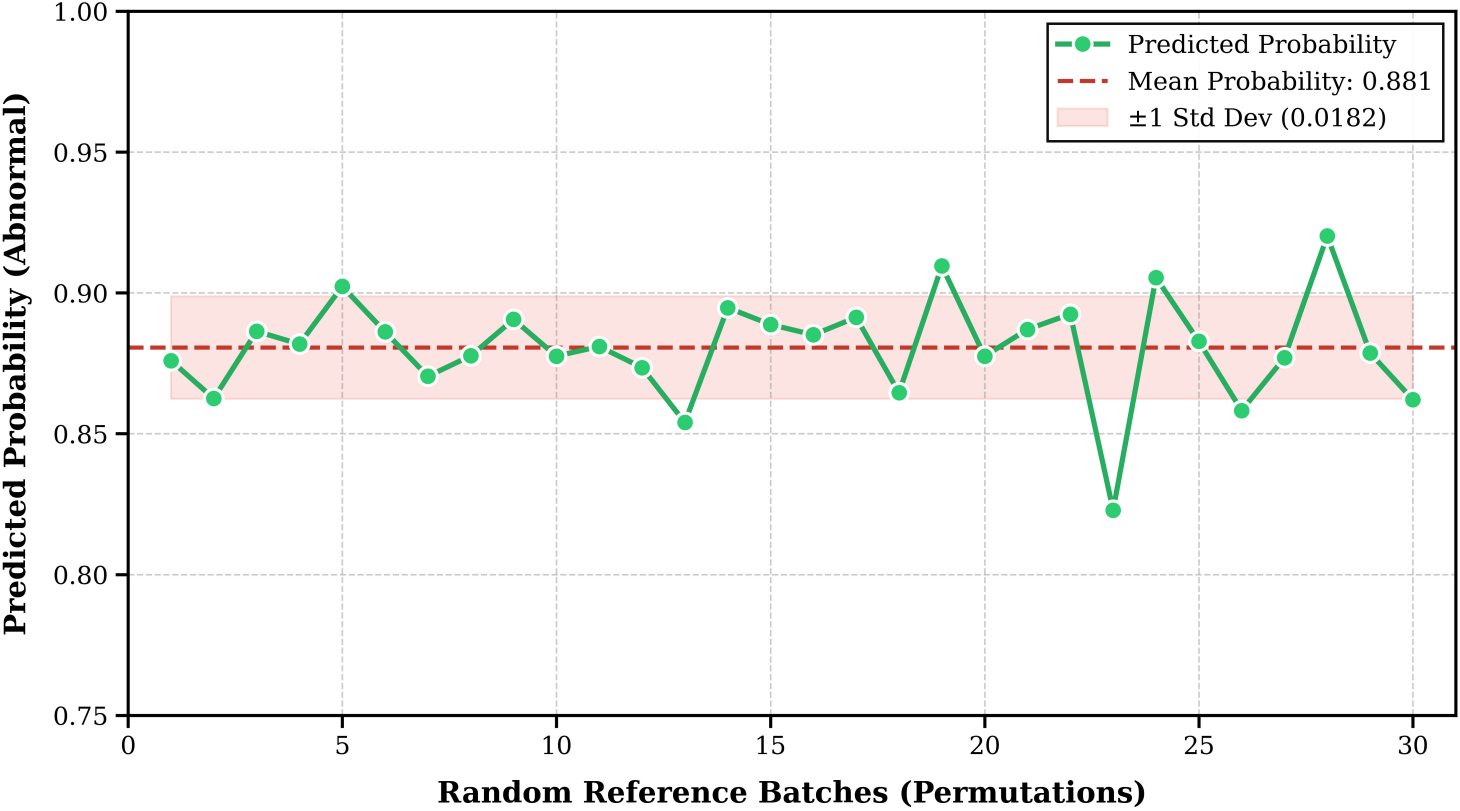
Inference stability analysis against neighbor variation. To evaluate sensitivity to reference batch composition, we tracked predicted probabilities of a representative diseased subject across 30 random trials. The prediction demonstrates high stability (μ = 0.881, σ = 0.0182), proving the robustness of the implicit interaction mechanism.

As illustrated in Figure 5, the predicted probability for the abnormal subject remained consistently high and stable, yielding a mean probability (μ) of 0.881 with a minimal standard deviation (σ) of 0.0182. This empirical behavior highlights two key properties. First, the extremely low variance confirms that the IP-Mamba module is not over-reliant on specific neighbors, effectively filtering out spurious correlations from the batch context. Second, the stability ensures that the final diagnostic decision is highly reliable. Even with variations in the reference batch, the predicted probability remains consistently above the decision threshold (τ = 0.5). This suggests that IP-Mamba provides consistent screening outcomes across diverse clinical batch environments, aligning with the rigorous robustness standards required for reliable medical diagnostics (An et al., 2020).

## 4 Conclusion

In this paper, we introduced IP-Mamba, a computationally efficient framework that advances population-based neuroimaging analysis. By shifting from explicit, computationally expensive *O*(*N*^2^) graph constructions to an implicit, set-based state space modeling approach, we effectively bridge the high-dimensional complexity of 3D neuroimaging data with the clinical requirements for population-level reasoning. Embodying a design-driven philosophy of “participatory intelligence,” our framework successfully translates individual feature extraction into a collective diagnostic consensus, mimicking the collaborative diagnostic process of human medical experts.

The core novelty of our approach lies in the integration of a Bidirectional Shared Mamba mechanism with a Shuffle Consistency Strategy (ℒ_*cons*_). This architectural design substantially resolves the fundamental theoretical conflict between the sequential processing inherent in state space models and the unordered nature of subject cohorts. Consequently, IP-Mamba achieves effective permutation equivariance and highly robust inference stability, ensuring that diagnostic decisions remain objective regardless of the reference batch composition.

Empirical validations on the OASIS-1 dataset for binary clinical screening confirm the superiority of our framework. By initializing a 3D-ResNet50 backbone with large-scale medical pre-training and leveraging latent population context, IP-Mamba emerges as a competitive population-aware alternative to standard 3D-CNN and ViT baselines, achieving a highly competitive Balanced Accuracy of 87.84% and a high Recall of 0.89 for the diseased cohort. Crucially, IP-Mamba maintains an *O*(*N*) linear memory footprint, overcoming the quadratic memory explosion typical of Graph Transformers. This structural efficiency ensures that high-resolution population modeling is safely deployable on high-memory consumer GPUs, effectively mitigating hardware barriers in limited clinical cohorts.

Looking forward, IP-Mamba establishes a robust foundation as a specialized Implicit Population Learner tailored for neuroimaging analysis. While the current validation focuses on structural MRI, the framework’s linear scalability and robust set-modeling capabilities open new avenues for future research. It holds immense potential for integrating heterogeneous clinical modalities—such as genetics, demographic data, and cognitive scores—into a unified, interaction-aware decision support system for the early diagnosis of neurodegenerative diseases. Furthermore, we plan to investigate SSM-specific interpretability techniques, such as state perturbation analysis, in future studies to provide more granular feature attribution.

## Data Availability

Publicly available datasets were analyzed in this study. This data can be found at: Repository name: OASIS-1 (Open Access Series of Imaging Studies). Direct link: https://www.oasis-brains.org/.
